# Development and initial validation of a family activation measure for acute care

**DOI:** 10.1371/journal.pone.0286844

**Published:** 2024-01-31

**Authors:** Sophie Hallot, Vanessa Debay, Nadine Foster, Karen E. A. Burns, Michael Goldfarb

**Affiliations:** 1 McGill Faculty of Medicine, McGill University, Montreal, QC, Canada; 2 Patient and Family Partnership Committee, Canadian Critical Care Trials Group, Markham, ON, Canada; 3 Division of Critical Care, University of Calgary, Calgary, AB, Canada; 4 Interdepartmental Division of Critical Care, Department of Medicine, University of Toronto, Toronto, Canada; 5 Li Ka Shing Knowledge Institute, St. Michael’s Hospital, Toronto, Canada; 6 Department of Health Research Methods, Evidence, and Impact, McMaster University, Hamilton, Canada; 7 Division of Cardiology, Jewish General Hospital, McGill University, Montreal, QC, Canada; University of Sharjah College of Health Sciences, UNITED ARAB EMIRATES

## Abstract

**Background:**

Activation of a family member refers to their desire, knowledge, confidence, and skills that can inform engagement in healthcare. Family activation combined with opportunity can lead to engagement in care. No tool currently exists to measure family activation in acute care. Therefore, we aimed to develop and validate a tool to measure family activation in acute care.

**Methods:**

An interdisciplinary team of content experts developed the FAMily Activation Measure (FAM-Activate) through an iterative process. The FAM-Activate tool is a 4-item questionnaire with 5 Likert-type response options (ranging from strongly agree to strongly disagree). Scale scores are converted to a 0–100 point scoring range so that higher FAM-Activate scores indicate increased family activation. An overall FAM-Activate score (range 0–100) is calculated by adding the scores for each item and dividing by 4. We conducted reliability and predictive validity assessments to validate the instrument by administering the FAM-Activate tool to family members of patients in an acute cardiac unit at a tertiary care hospital. We obtained preliminary estimates of family engagement and satisfaction with care.

**Results:**

We surveyed 124 family participants (age 54.1±14.4; 73% women; 34% non-white). Participants were predominantly the adult child (38%) or spouse/partner (36%) of patients. The mean FAM-Activate score during hospitalization was 84.1±16.1. FAM-Activate had acceptable internal consistency (Cronbach’s *a* = 0.74) and showed test-retest responsiveness. FAM-Activate was moderately correlated with engagement behavior (Pearson’s correlation r = 0.47, P <0.0001). The FAM-Activate score was an independent predictor of family satisfaction, after adjusting for age, gender, relationship, and living status.

**Conclusion:**

The FAM-Activate tool was reliable and had predictive validity in the acute cardiac population. Further research is needed to explore whether improving family activation can lead to improved family engagement in care.

## Introduction

**Fig 1:**
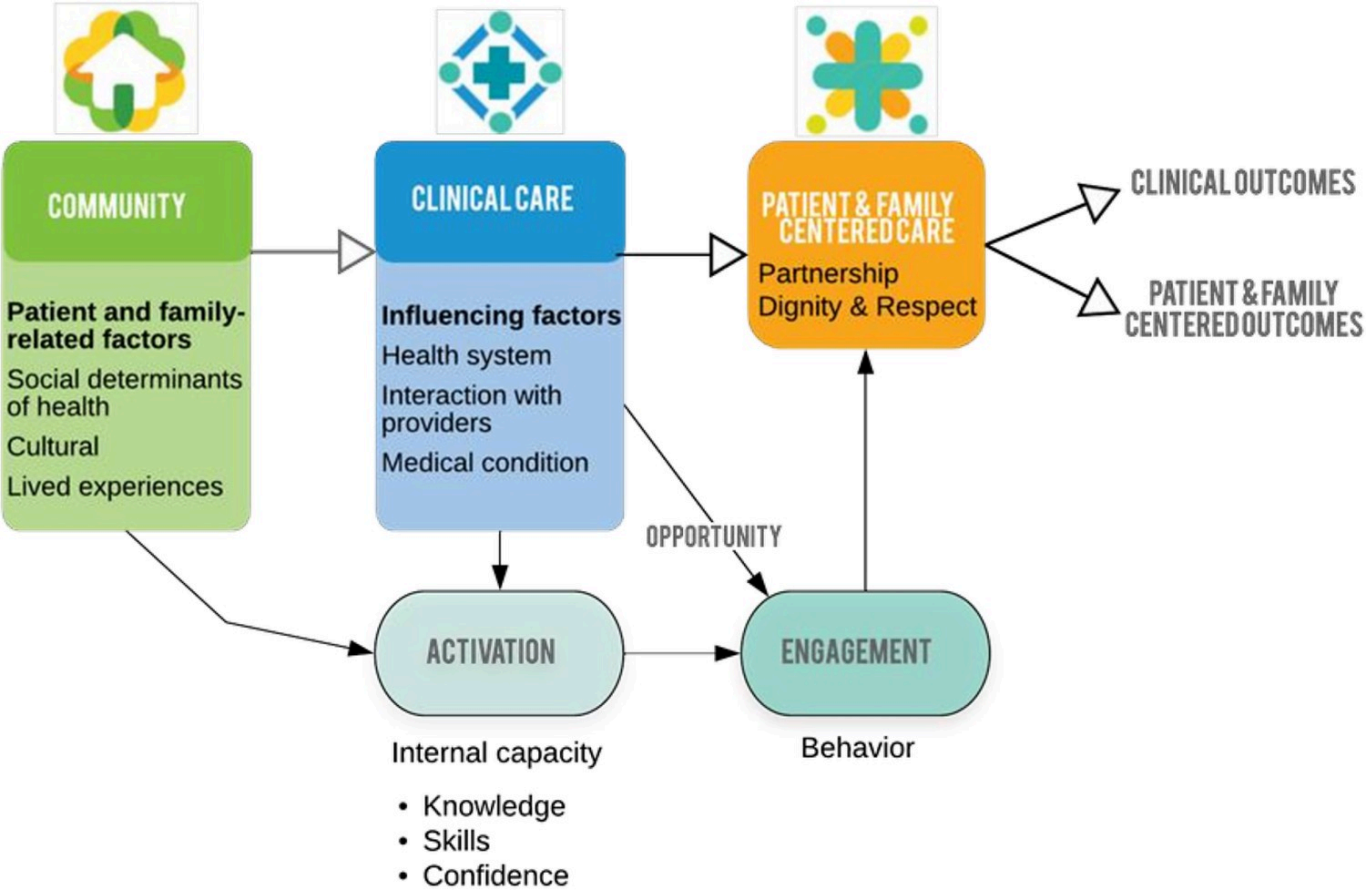
Patient and family-centered care overview.

The activation of a family member is the desire, knowledge, confidence, and skills that family members possess. Activation is informed by patient- and family-related factors such as personal attributes, social determinants of health, culture, and lived experiences [1]. Activation may be further influenced by interactions and experiences within the healthcare system. Family activation combined with opportunity can lead to engagement in care **(Fig 1)**. Family engagement is the means to achieve patient- and family-centered care, which is a goal of contemporary healthcare delivery and is associated with improved family care satisfaction and psychological symptoms [2, 3].

Hibbard and colleagues first operationalized the concept of activation in the healthcare context for patients with chronic illness nearly two decades ago [4]. The impetus for developing this operational model was the emergence of patient-oriented care and involvement of healthcare consumers as informed participants in care. More recently, there has been an increasing recognition of the important role of the family member as a key participant in the healthcare process [5]. Family members may participate in numerous areas of patient care, including patient advocacy, surrogate decision making, physical and emotional support, treatment adherence, and even direct care provision. Exploring the role of family activation is necessary to understand the capacity for family members to participate in their loved one’s care. Quantifying family activation can inform whether engagement strategies can improve family skills, knowledge, and confidence in their ability to participate in care.

There is a need to explore how interactions between the family and the healthcare team can modify the family activation level. It is important to understand how family activation and its’ component principles (desire, knowledge, confidence, and skills) are associated with engagement behavior. Family activation may also predict of posthospital discharge family-centered outcomes, such as care satisfaction and mental health. Although there is a strong need to develop and validate a tool to measure activation, no tool currently exists. We aimed to develop and validate a tool to measure family activation in acute care.

## Methods

### Development of the family activation measure

The FAMily Activation tool (FAM-Activate) was developed and tested using the method described by Hamzeh and colleagues [6]. An interdisciplinary team of content experts developed the FAMily Activation Measure (FAM-Activate) through an iterative process. Our interdisciplinary group included experts in person-centered care, questionnaire development, acute clinical care, and patient- and family partnership.

### Literature review

We reviewed the published and grey literature for tools evaluating family activation of families in the acute healthcare context. To identify relevant studies, we searched the literature from 1980 to 2022 using the following combinations of keywords: family (family, family member, caregiver), activation (desire, knowledge, skills, confidence), and measurement (measure, instrument, tool).

### Tool development process

Questionnaire items were generated by interdisciplinary team members using the key activation concepts: desire, knowledge, confidence, and skills as questionnaire domains [7]. In developing the questionnaire, we sought to capture the four stages of activation as outlined by Hibbard and colleagues: Level 1, believes in the importance of active role; Level 2, confidence and knowledge to take action; Level 3, taking action; Level 4, staying the course under stress [4]. In this framework, higher levels reflect increased levels of activation. In item generation, we endeavored to include at least 1-item for each concept. In item reduction, we reduced the number of potential items for inclusion in each domain while retaining the most salient items to reduce respondent burden [6]. The initial version of the questionnaire included eight items with approximately two items per domain. After further discussion and testing, the questionnaire version used for testing was reduced to 4 items total, with 1 item for each level and domain (**S1 Table**).

We pilot-tested the questionnaire with a nurse educator, nurse manager, quality-of-care expert, two patient/family partners, and two members of the general public to identify poorly worded or irrelevant items. We revised the questionnaire after each phase of testing based on respondent feedback. In clinical sensibility testing, the questionnaire was reviewed by the patient/family partners and two physicians with expertise in patient- and family-centered care. The questionnaire was translated into both official languages (English and French). The final version of the questionnaire was formatted at an 8th-grade reading level using the Flesch-Kincaid Grade Level Index [8].

### Questionnaire composition and scoring

FAM-Activate is composed of 4 items with each item rated on a five-point Likert scale (1 = strongly agree, 2 = agree, 3 = neutral, 4 = disagree, 5 = strongly disagree). Scale scores are then changed to a 0–100-point scoring range. For example, a scale score of 1 (strongly agree) would be changed to 100, a scale score of 2 (agree) would be changed to 75, and so on. Higher scores indicate greater family activation. An overall FAM-Activate score (range 0–100) is calculated by adding the scores for each item and dividing by 4.

### Study design

We performed a prospective observational study in the acute cardiac unit of the Jewish General Hospital, an academic tertiary care center in Montreal, Canada. The institutional quality improvement (QI) department approved the study as a QI project. Research ethics approval and informed consent was waived for this study. The study was reported according to the COSMIN reporting guidelines for patient-reported outcome measures [9].

### Study procedures

We administered the FAM-Activate questionnaire to family members of patients admitted in the acute cardiac unit. Between May 2022 to August 2022, study personnel administered the questionnaire on 14 non-consecutive days over four months (May 2022 to August 2022). On questionnaire distribution days, study personnel systematically approached each patient room in the cardiac care unit. If no family member was present, study personnel returned at least once during the same time period (morning, afternoon, or evening) to check to see if a family member was present. We considered anyone whom the patient would like to be involved in their care to be ‘family’ [10]. Family members were eligible to participate if they could understand English or French and were 18 years of age or older. The potential family member participant was determined using the following approach: Patients who were capable of communicating were asked to identify the family member whom they would like to be approached. For patients who were not capable of communicating, the designated contact person was eligible, or if unavailable, the family member present at the bedside. Only one family member per patient was eligible to complete the questionnaire.

### Data collection procedures

Study personnel obtained sociodemographic information and administered the FAM-Activate and FAMily Engagement (FAME) questionnaires. The FAME questionnaire comprises 12 items and includes the domains testing family presence, communication/education, decision-making, care contribution, and family needs. FAME has been validated in an acute care context [11]. We collected the following sociodemographic information for each participant: age, gender, relationship to the patient, race/ethnicity, living status (living with or not living with the patient), and highest level of education achieved.

Subsequently, we contacted participating family members within two weeks following hospital discharge by phone or email to complete the Family Satisfaction in the Intensive Care Unit (FS-ICU) instrument. The FS-ICU instrument is a validated and widely used tool to assess family satisfaction with care in both the intensive care and acute care unit settings [11, 12]. The tool consists of 24 questions with items pertaining to patient- and family-centered care. Over a two-week study period, study personnel also administered FAM-Activate to participants following discharge to test the responsiveness of the instrument.

### Statistical analyses

The sample size required for a validation study depends on several elements, including the number of factors and variables [13]. As a rule of thumb, validation studies generally require at least 10 respondents for each scale item (i.e., a 10:1 ratio) [14]. There were 4 scale items in our questionnaire, which computes to a sample size of 40 participants. However, we endeavored to include at least 100 participants to allow for analysis of impact of key variables (i.e., age, gender, relationship) on the activation score.

Continuous data are presented as mean ± standard deviation and between-group differences were tested with the student’s t-test or one-way analysis of variance, as appropriate. Categorical data are presented as frequencies and percentages and compared using the chi-squared test or the Fisher exact test, as appropriate. Mean FAM-Activate scores were compared between each pair of items in ascending order (i.e., Q1 to Q2; Q2 to Q3) using the one-sample t-test and across items (Q1, Q2, Q3, Q4) using one-way analysis of variance (ANOVA).

We assessed internal consistency testing using Cronbach’s alpha to assess the reliability of FAM-Activate. The Cronbach alpha is interpreted as excellent for an alpha of 0.91–1.0, good for an alpha of 0.81–0.90, good and acceptable for an alpha of 0.71–0.80, acceptable for an alpha of 0.61–0.70, and non-acceptable for an alpha of 0.01–0.60 [15].

We used a multivariable linear regression model adjusted for selected participant characteristics to evaluate the relationship between family activation and family care satisfaction (FS-ICU). We used Pearson’s correlation to assess the relationship between activation (total FAM-Activate score) and engagement (FAME score).

To test instrument responsiveness, we used the one-sample t-test to compare mean FAM-Activate scores on enrolment and post-discharge. To explore whether there were biases in the missing data, sensitivity analyses were performed to compare baseline demographics (age, gender), mean FAM-Activate scores and FAME scores for participants with and without FS-ICU scores available. All P-values were two-sided with values ≤ 0.05 indicating statistical significance. Statistical analyses were performed using SPSS 26.0 statistical software (IBM Corp, Armonk, New York).

## Results

### Cohort characteristics

We surveyed 124 family members. The mean age was 54.1±14.4 years. There were 90 (72.6%) women, and 44 (33.9%) family members were non-white (**Table 1**). Adult children (47 (37.9%)) and spouse/partner (45 (36.3%)) were the most common familial relationships to patients More than half of family members lived with the patient (59; 47.6%) and three-quarters of participants had post-secondary education (94; 75%). There were minimal missing data for baseline patient characteristics (race/ethnicity was missing for 2.4% of participants and level of education for 1.6% of participants).

**Table 1:** Demographics characteristics of family participants.

Participant characteristics	N = 124
**Age**, years	54.1 ± 14.4
**Gender**	
Man	34 (27.4%)
Woman	90 (72.6%)
Other	0 (0%)
**Relationships**	
Spouse/Partner	45 (36.3%)
Parent	2 (1.6%)
Daughter/son	47 (37.9%)
Sister/ brother	14 (11.3%)
Friend or neighbor	4 (3.2%)
Other	12 (9.7%)
**Race/Ethnicity**	
White (non-Hispanic)	80 (64.5%)
White (Hispanic)	3 (2.4%)
Black	4 (3.2%)
Asian	17 (13.7%)
Indigenous	2 (1.6%)
Other	15 (12.1%)
Missing	3 (2.4%)
**Lives with patient**	
Yes	59 (47.6%)
No	65 (52.4%)
**Level of education**	
Did not complete high school	11 (8.9%)
Completed high school	18 (14.5%)
Post-secondary program (not university)	39 (31.5%)
University degree	37 (29.8%)
Graduate degree	17 (13.7%)
Missing	2 (1.6%)

Abbreviations: Data are displayed as frequencies (percentage) or mean (standard deviation)

### FAM-Activate scores

**Fig 2:**
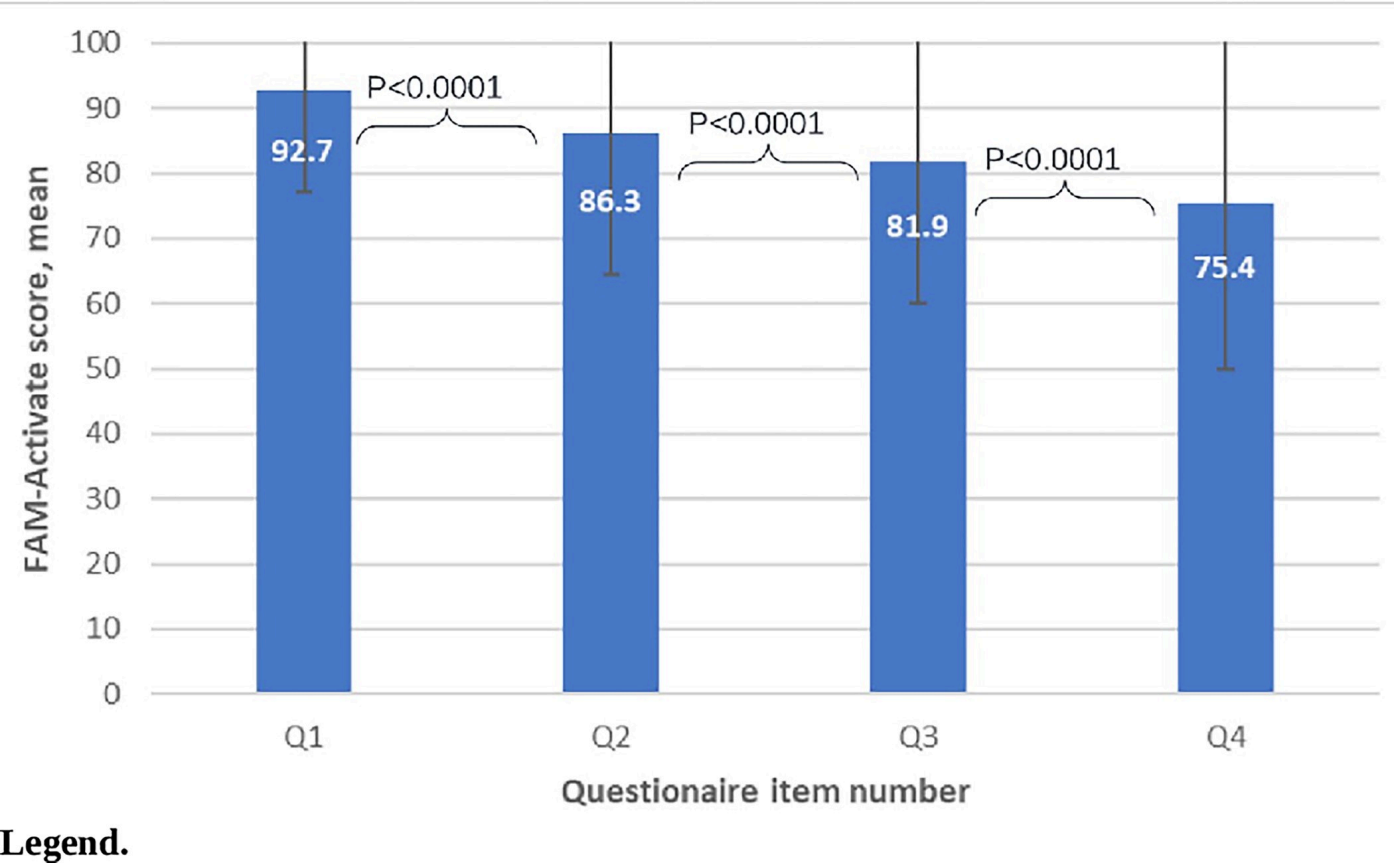
Mean FAM-Activate score by questionnaire item number. Standard deviation bars are included for each value.

The mean FAM-Activate score was 84.1±16.1 (**S1 Fig**). FAM-Activate item mean scores decreased sequentially from the first to the last item (P<0.0001; **Fig 2**). FAM-Activate scores were similar by age (dichotomized to greater or less than 65 years old), race, living status, and educational level (**S2 Table**). FAM-Activate scores differed by gender, with men having higher FAM-Activate scores than women (86.3±12.5 vs. 78.3±22.4, P = 0.01, respectively).

Twenty-three participants also had FAM-Activate administered after hospital discharge. The mean FAM-Activate score in this group increased from 84.8±15.4 to 89.3±14.9 (P<0.0001).

### Validation testing

The FAM-Activate tool had acceptable internal consistency (Cronbach’s *a* = 0.74). FAM-Activate was moderately correlated with engagement behavior (Pearson’s correlation, r = 0.47, P <0.0001).

In the multivariable analysis, both gender and FAM-Activate were independent predictors of family satisfaction (P = 0.04; **Table 2**). Each of the four items in FAM-Activate correlated with the FAME score but not the FS-ICU score (**S3 Table**).

**Table 2:** Multivariable analysis of predictors of family satisfaction in the ICU score.

Variable	P-value
Age	0.33
Gender	0.04
Relationship	0.31
Living status	0.44
FAM-Activate	0.04

## Discussion

The study aimed to describe the development of a family activation measure and to validate its use in an acute cardiac care setting. We found that FAM-Activate had face validity, reliability, and predictive validity in an acute cardiac care population. FAM-Activate scores were positively correlated with family engagement behavior during hospitalization. Moreover, FAM-Activate scores independently predicted family satisfaction scores post-hospital stay. To our knowledge, this is the first validated instrument to measure family activation in the acute care setting.

Several tools to measure activation have been developed for caregivers of people with chronic illnesses. The Caregiver-Patient Activation Measure (CG-PAM) is a 10-item instrument designed to measure activation in caregivers to another adult with a long-term or chronic illness [16]. CG-PAM had reasonable reliability, but other validation measures showed inconsistent findings. Managing Your Loved One’s Health (MYLOH) is a 29-item instrument validated for primary caregivers (n = 190) of individuals with advanced chronic illnesses and focused on identifying gaps in caregivers’ knowledge, skills, and access to support [17]. Partnering for Better Health—Living with Chronic Illness: Dementia (PBH-LCI:D) is a 32-item validated instrument focused on measuring activation in caregivers of people with dementia in the longitudinal care setting [18].

There is a strong need for an instrument to measure family activation in the acute care setting. Family involvement in acute care differs from the outpatient setting. De novo disease presentation may thrust family members into the caregiver role for the first time or necessitating a greater degree of care involvement than previously required. In acute care, opportunities exist for family members to be involved in engagement behaviors such as participation in team rounds, family presence during procedures, interdisciplinary care conferences, and direct care delivery (i.e., mobilization and delirium detection) [19]. In addition, the hospitalization of a loved one can be a stressful experience for family members and have a considerable psychological impact. Family members may find themselves navigating a busy, unfamiliar environment, and often feel overwhelmed and unable to understand information about their loved one’s condition and management plan [20]. As a result, up to half of family members report depressive or anxiety symptoms in some settings, which often persist post-hospitalization [21]. Higher levels of caregiver depression, anxiety, and fatigue have been shown to be inversely related to caregiver activation [22]. At present, limited information exists to understand the relationship between caregiver activation and engagement in care and, ultimately, in patient- and family-centered outcomes in acute care [23]. Measuring family activation in acute care could assist healthcare organizations in evaluating efforts to improve family knowledge, skills, and motivation toward participation in care. In particular, FAM-Activate can be used to quantify the impact of innovative strategies on family activation in care. To this end, our tool may be further evaluated and assessed by future research to help characterize the relationship between family activation, engagement, and patient- and family-centered outcomes.

FAM-Activate was designed to be a pragmatic instrument. To reduce respondent burden while capturing all the domains of activation, only four items were included in the version of FAM-Activate studied. Even widely used tools, such as for the 36-Item Short Form Survey have been reconfigured (12-Item Short Form survey) to reduce respondent burden [24, 25]. Respondent fatigue to burdensome questionnaires is an important consideration when designing practical tools for acute care [26]. We sought to test whether only a limited number of items for each concept could still adequately assess the key conceptual domains of activation. To this end, we found that 4-Item FAM-Activate scores were correlated to family engagement in care and predictive of post-hospitalization care satisfaction. In addition, we structured the questionnaire structure so that higher scores reflected higher levels of activation and progressively higher levels of activation from questions 1 to 4. As anticipated, we found a stepwise reduction in item scores from the first to the fourth item. While each activation item was positively associated with engagement, we did not find a relationship between individual activation items and overall care satisfaction.

Family activation level is a potentially modifiable target for intervention. Activation is determined by a confluence of personal characteristics, sociodemographic considerations, lived experiences, and the influence of the healthcare context. There is a need to explore whether improving family activation leads to improved engagement in care and ultimately to improved clinical and patient- and family-centered outcomes. Interventions could be developed to improve specific domains of activation (i.e., skill to participate in certain care activities), which could result in higher levels of activation. Interventions could be tailored for specific ecological populations (i.e., Indigenous, Black, non-traditional family structures). There is also a need to validate the FAM-Activate in other care settings, such as the intensive care unit, and to further characterize how gender, ethnicity/race, relationship status, and sociodemographic factors affect activation. Measuring family activation during hospitalization could also allow QI teams to quantify the real-time impact of interventions targeting activation.

### Strengths and limitations

Strengths of this study include the use of rigorous methodology to develop, test, and administer the questionnaire, the engagement of a multidisciplinary team with expertise in the relevant domains, and reporting of our findings according to the COSMIN guideline. We recruited family members consecutively on questionnaire distribution days to reduce selection bias. We included patient/family advisors in the instrument development, testing, and validation process. The study cohort was diverse in terms of gender and racial/ethnic diversity. The main limitations of this preliminary study reflect its observational nature and that participants were recruited from a single cardiac care unit in an academic center. Future studies should include acutely ill populations from multiple sites.

## Conclusion

The FAM-Activate instrument was developed through an iterative process by content experts and end users to measure family activation in the acute care context. FAM-Activate was found to have face validity, reliability, and predictive validity in the population studied. Further studies are needed to assess FAM-Activate in other populations and to explore the relationship between activation, engagement, and patient- and family-centered outcomes.

## Supporting information

S1 Fig FAM-Activate total score distribution. (DOCX)

S1 Table FAM-Activate composition. (DOCX)

S2 Table Comparison of FAM-Activate scores. (DOCX)

S3 Table Relationship of engagement and satisfaction with individual FAM-Activate items. (DOCX)

## References

[1] Hibbard JH, Mahoney E. Toward a theory of patient and consumer activation. Patient Educ Couns. 2010;78(3):377–81. doi: 10.1016/j.pec.2009.12.015 20188505

[2] Agency for Healthcare Research and Quality. Guide to Patient and Family Engagement in Hospital Quality and Safety. 2021. https://www.ahrq.gov/patient-safety/patients-families/engagingfamilies/index.html. Accessed January 14, 2023.

[3] Goldfarb MJ, Bibas L, Bartlett V, Jones H, Khan N. Outcomes of Patient- and Family-Centered Care Interventions in the ICU: A Systematic Review and Meta-Analysis. Crit Care Med. 2017;45(10):1751–61. doi: 10.1097/CCM.0000000000002624 28749855

[4] Hibbard JH, Stockard J, Mahoney ER, Tusler M. Development of the Patient Activation Measure (PAM): conceptualizing and measuring activation in patients and consumers. Health services research. 2004;39(4 Pt 1):1005–26. doi: 10.1111/j.1475-6773.2004.00269.x 15230939 PMC1361049

[5] Goldfarb MJ, Bechtel C, Capers Qt, de Velasco A, Dodson JA, Jackson JL, et al. Engaging Families in Adult Cardiovascular Care: A Scientific Statement From the American Heart Association. J Am Heart Assoc. 2022:e025859. doi: 10.1161/JAHA.122.025859 35446109 PMC9238560

[6] Hamzeh J, Kaur N, Bush P, Hudon C, Schuster T, Vedel I, et al. Towards a comprehensive Questionnaire Origin and Development Appraisal tool: A literature review and a modified nominal group. Education for Information. 2019;35:7–20.

[7] Von Korff M, Gruman J, Schaefer J, Curry SJ, Wagner EH. Collaborative Management of Chronic Illness. Annals of Internal Medicine. 1997;127(12):1097–102. doi: 10.7326/0003-4819-127-12-199712150-00008 9412313

[8] Kincaid JP, Fishburne RP, Rogers RL, Chissom BS, editors. Derivation of New Readability Formulas (Automated Readability Index, Fog Count and Flesch Reading Ease Formula) for Navy Enlisted Personnel. 1975.

[9] Gagnier JJ, Lai J, Mokkink LB, Terwee CB. COSMIN reporting guideline for studies on measurement properties of patient-reported outcome measures. Qual Life Res. 2021;30(8):2197–218. doi: 10.1007/s11136-021-02822-4 33818733

[10] Brown SM, Rozenblum R, Aboumatar H, Fagan MB, Milic M, Lee BS, et al. Defining patient and family engagement in the intensive care unit. Am J Resp Crit Care Med. 2015;191(3):358–60. doi: 10.1164/rccm.201410-1936LE 25635496

[11] Hallot S DV, Debigaré S, Foster N, Soboleva N, Burns K, Goldfarb M. Validation of an Instrument to Measure Family Engagement in Acute Cardiac Care. CJC Open. 2023:1–7. 37013077 10.1016/j.cjco.2022.11.021 PMC10066429

[12] Wall RJ, Engelberg RA, Downey L, Heyland DK, Curtis RJ. Refinement, scoring, and validation of the Family Satisfaction in the Intensive Care Unit (FS-ICU) survey*. Critical Care Medicine. 2007;35(1). doi: 10.1097/01.CCM.0000251122.15053.50 17133189

[13] Boateng GO, Neilands TB, Frongillo EA, Melgar-Quiñonez HR, Young SL. Best Practices for Developing and Validating Scales for Health, Social, and Behavioral Research: A Primer. Front Public Health. 2018;6:149. doi: 10.3389/fpubh.2018.00149 29942800 PMC6004510

[14] MacCallum RC, Widaman KF, Zhang S, Hong SJPm. Sample size in factor analysis. 1999;4(1):84. 10.1207/S15327906MBR3604_06 26822184

[15] Cortina JM. What is coefficient alpha? An examination of theory and applications. Journal of Applied Psychology. 1993;78(1):98–104.

[16] Carleton-Eagleton K, Walker I, Gibson D, Freene N, Semple S. Testing the validation and reliability of the Caregiver-Patient Activation Measure (CG-PAM). PEC Innovation. 2022;1:100098. doi: 10.1016/j.pecinn.2022.100098 37213753 PMC10194270

[17] Borson S, Mobley P, Fernstrom K, Bingham P, Sadak T, Britt HR. Measuring caregiver activation to identify coaching and support needs: Extending MYLOH to advanced chronic illness. PloS one. 2018;13(10):e0205153–e. doi: 10.1371/journal.pone.0205153 30307980 PMC6181336

[18] Sadak T, Korpak A, Borson S. Measuring caregiver activation for health care: Validation of PBH-LCI:D. Geriatr Nurs. 2015;36(4):284–92. doi: 10.1016/j.gerinurse.2015.03.003 25959036

[19] Goldfarb M, Bibas L, Burns K. Patient and Family Engagement in Care in the Cardiac Intensive Care Unit. Can J Cardiol. 2020;36(7):1032–40. doi: 10.1016/j.cjca.2020.03.037 32533931

[20] Gill M, Bagshaw SM, McKenzie E, Oxland P, Oswell D, Boulton D, et al. Patient and Family Member-Led Research in the Intensive Care Unit: A Novel Approach to Patient-Centered Research. PLoS One. 2016;11(8):e0160947. doi: 10.1371/journal.pone.0160947 27494396 PMC4975402

[21] Anderson WG, Arnold RM, Angus DC, Bryce CL. Posttraumatic stress and complicated grief in family members of patients in the intensive care unit. J Gen Intern Med. 2008;23(11):1871–6. doi: 10.1007/s11606-008-0770-2 18780129 PMC2585673

[22] Hetland BD, McAndrew NS, Kupzyk KA, Krutsinger DC, Turnbull AE, Pozehl BJ, et al. Relationships among Demographic, Clinical, and Psychological Factors Associated with Family Caregiver Readiness to Participate in Intensive Care Unit Care. Ann Am Thorac Soc. 2022;19(11):1881–91. doi: 10.1513/AnnalsATS.202106-651OC 35649201

[23] Dingley CE, Clayton M, Lai D, Doyon K, Reblin M, Ellington L. Caregiver Activation and Home Hospice Nurse Communication in Advanced Cancer Care. Cancer Nursing. 2017;40(5). doi: 10.1097/NCC.0000000000000429 27631112 PMC5830116

[24] Ware JE Jr, Sherbourne CD. The MOS 36-item short-form health survey (SF-36). I. Conceptual framework and item selection. Medical care. 1992;30(6):473–83. 1593914

[25] Ware J Jr, Kosinski M, Keller SD. A 12-Item Short-Form Health Survey: construction of scales and preliminary tests of reliability and validity. Medical care. 1996;34(3):220–33. doi: 10.1097/00005650-199603000-00003 8628042

[26] O’Reilly-Shah VN. Factors influencing healthcare provider respondent fatigue answering a globally administered in-app survey. PeerJ. 2017;5:e3785. doi: 10.7717/peerj.3785 28924502 PMC5600176

