# Supplementary material for: Development and initial validation of a family activation measure for acute care
Source: PLoS One. 2024 Jan 31;19(1):e0286844. doi: 10.1371/journal.pone.0286844 (PMC10830022; doi:10.1371/journal.pone.0286844)
Supplement: S1 Fig — (DOCX) [file pone.0286844.s001.docx]

**Figure S1.** FAM-Activate total score distribution
