# Supplementary material for: Development and initial validation of a family activation measure for acute care
Source: PLoS One. 2024 Jan 31;19(1):e0286844. doi: 10.1371/journal.pone.0286844 (PMC10830022; doi:10.1371/journal.pone.0286844)
Supplement: S1 Table — (DOCX) [file pone.0286844.s002.docx]

**Table S1**. FAM-Activate composition

| **Item** | **Text** | **Domains** | **Activation level** |
| --- | --- | --- | --- |
| **1** | I want to be actively involved in managing my family member’s care | Desire | 1 |
| **2** | I understand my family member’s health condition | Knowledge | 2 |
| **3** | I know how to be involved in my family member’s health care | Confidence  Knowledge | 3 |
| **4** | I can figure out solutions when problems occur with my family member’s health condition | Confidence  Skills | 4 |
