# Supplementary material for: Development and initial validation of a family activation measure for acute care
Source: PLoS One. 2024 Jan 31;19(1):e0286844. doi: 10.1371/journal.pone.0286844 (PMC10830022; doi:10.1371/journal.pone.0286844)
Supplement: S2 Table — (DOCX) [file pone.0286844.s003.docx]

**Table S2**. Comparison of FAM-Activate scores

| **Participant characteristics** | **FAM-Activate score** | **P-value** |
| --- | --- | --- |
| **Age**  Age ≥ 65 (N=93)  Age < 65 (N=31) | 83.9±12.9  83.9±17.2 | 0.99 |
| **Gender**  Man (N=34)  Woman (N=90)  Other (N=0) | 86.3±12.5  78.3±22.4 | 0.01 |
| **Relationships**  Spouse/Partner (N=45)  Parent (N=2)  Daughter/son (N=47)  Sister/brother (N=14)  Friend or neighbor (N=4)  Other (N=12) | 87.5±14.7  100.0±0.0  83.6±13.9  76.3±22.4  81.3±8.8  80.7±20.9 | 0.16 |
| **Race**  White (non-Hispanic) (N=80)  Non-white (N=31)  Missing (N=3) | 84.2±15.7  83.6±17.5  90.6±4.4 | 0.83 |
| **Living Together**  Yes (N=59)  No (N=65) | 86.3±16.8  82.1±15.3 | 0.15 |
| **Level of education**  Did not complete high school (N=11)  Completed high school (N=18)  Post-secondary program; not university (N=39)  University degree (N=37)  Graduate degree (N=17) | 78.4 ± 22.2  86.8 ± 8.5  82.4±17.0  86.5± 13.1  82.4±21.7 | 0.47 |
