# Supplementary material for: Development and initial validation of a family activation measure for acute care
Source: PLoS One. 2024 Jan 31;19(1):e0286844. doi: 10.1371/journal.pone.0286844 (PMC10830022; doi:10.1371/journal.pone.0286844)
Supplement: S3 Table — (DOCX) [file pone.0286844.s004.docx]

**Table S3**. Relationship of engagement and satisfaction with individual FAM-Activate items

|  | **Pearsons correlation coefficient** | **P-value** |
| --- | --- | --- |
| Q1 score and FAME | 0.31 | <0.001 |
| Q2 score and FAME | 0.28 | 0.001 |
| Q3 score and FAME | 0.48 | <0.001 |
| Q4 score and FAME | 0.34 | <0.001 |
| Q1 score and FSICU | 0.07 | 0.66 |
| Q2 score and FSICU | 0.25 | 0.13 |
| Q3 score and FSICU | 0.24 | 0.14 |
| Q4 score and FSICU | 0.28 | 0.08 |

**Legend**.

Abbreviations: FAME, FAMily Engagement; FSICU, Family Satisfaction in the Intensive Care Unit
